# Cheiradone: a vascular endothelial cell growth factor receptor antagonist

**DOI:** 10.1186/1471-2121-9-7

**Published:** 2008-01-29

**Authors:** Sajjad Hussain, Mark Slevin, Mohammad A Mesaik, Mohammad I Choudhary, Abdul H Elosta, Sabine Matou, Nessar Ahmed, David West, John Gaffney

**Affiliations:** 1 School of Biology, Chemistry and Health Science, Manchester Metropolitan University, Chester St. Manchester M1 5GD, UK; 2 School of Biological Sciences, University of Liverpool, Liverpool, L69 7ZB, UK; 3 H.E.J. Research Institute of Chemistry, International Centre for Biological and Chemical Sciences, University of Karachi, Karachi 75720, Pakistan

## Abstract

**Background:**

Angiogenesis, the growth of new blood vessels from the pre-existing vasculature is associated with physiological (for example wound healing) and pathological conditions (tumour development). Vascular endothelial growth factor (VEGF), fibroblast growth factor-2 (FGF-2) and epidermal growth factor (EGF) are the major angiogenic regulators. We have identified a natural product (cheiradone) isolated from a *Euphorbia *species which inhibited *in vivo *and *in vitro *VEGF- stimulated angiogenesis but had no effect on FGF-2 or EGF activity. Two primary cultures, bovine aortic and human dermal endothelial cells were used in *in vitro *(proliferation, wound healing, invasion in Matrigel and tube formation) and *in vivo *(the chick chorioallantoic membrane) models of angiogenesis in the presence of growth factors and cheiradone. In all cases, the concentration of cheiradone which caused 50% inhibition (IC_50_) was determined. The effect of cheiradone on the binding of growth factors to their receptors was also investigated.

**Results:**

Cheiradone inhibited all stages of VEGF-induced angiogenesis with IC_50 _values in the range 5.20–7.50 μM but did not inhibit FGF-2 or EGF-induced angiogenesis. It also inhibited VEGF binding to VEGF receptor-1 and 2 with IC_50 _values of 2.9 and 0.61 μM respectively.

**Conclusion:**

Cheiradone inhibited VEGF-induced angiogenesis by binding to VEGF receptors -1 and -2 and may be a useful investigative tool to study the specific contribution of VEGF to angiogenesis and may have therapeutic potential.

## Background

Angiogenesis, the growth of new blood vessels from the existing vasculature is associated with physiological (wound healing, endometrial cycle and embryonic development) and pathological processes (tumour growth, rheumatoid arthritis, diabetic retinopathy, and brain and cardiac infarctions) [1,2]. Angiogenesis is mediated by pro-angiogenic factors including vascular endothelial cell growth factor (VEGF), fibroblast growth factor-2 (FGF-2), angiopoietin, and epidermal growth factor (EGF) [3-6].

VEGF comprises a family of multifunctional cytokines which include the variants VEGF-A, -B, -C, -D and-E and placental growth factor (PlGF) [7,8]. VEGF-A is mitogenic *in vitro *and angiogenic *in vivo *[9,10] and its role in angiogenesis and vasculogenesis has been elucidated [11-13]. At least nine different isoforms of human VEGF-A have been identified with 121, 145, 148, 162, 165, 183, 189 and 206 amino acid residues [14,8]. Of these, VEGF_165 _is most clearly associated with pathological angiogenesis [8] and exerts its biological action upon binding with two high affinity receptor tyrosine kinases; VEGFR-1 (flt-1) and VEGFR-2 (kinase domain receptor; flk-1) [8,15]. The role of these receptors, especially flk-1 in angiogenesis has been confirmed through gene knockout studies and flk-1^-/- ^embryos are unable to form blood islands and to generate haematopoietic precursors [reviewed in [16]]. VEGFR-1 has a 50 times higher binding affinity for VEGFR-1 than VEGFR-2 [17] however, VEGFR-2 has a stronger receptor tyrosine kinase activity than VEGFR-1 and acts as a major mitogenic receptor on endothelial cells (ECs) [16,18].

Due to the central role of angiogenesis in tumour growth and progression it has been a target in cancer therapy. For example Bevacizumab, a VEGF-A blocking antibody has been approved for the treatment of metastatic colorectal cancer [19] and Sunitinib, a VEGF receptor antagonist for treatment of gastrointestinal stromal tumours and for advanced renal cell carcinoma [20]. Several other VEGF inhibitors including the receptor tyrosine kinase inhibitors (RTKIs), Pegaptanib and Sorafenib have been tested in phase-1 to phase-III clinical trials against VEGF-associated malignancies [21,22].

Natural compounds from medicinal plants display diverse pharmacological activities [23] and have advantages over synthetic drugs, such as smoother action, better tolerance and fewer allergic reactions. Cheiradone, a naturally occurring plant diterpene, was isolated from the medicinal plant *Euphobia chiradenia *and in preliminary screening was shown to be a PLA_2 _inhibitor, have anti-inflammatory properties and inhibit wound healing although the mechanisms of action were not investigated [24].

In this study we have investigated the effect of cheiradone on VEGF-induced angiogenesis and show VEGF_165 _binding to VEGFR-1 and -2 resultined in inhibition of *in vitro *and *in vivo *angiogenesis.

## Results

### Cheiradone inhibited VEGF_165 _binding to VEGFR-1 and -2

**Figure 1:**
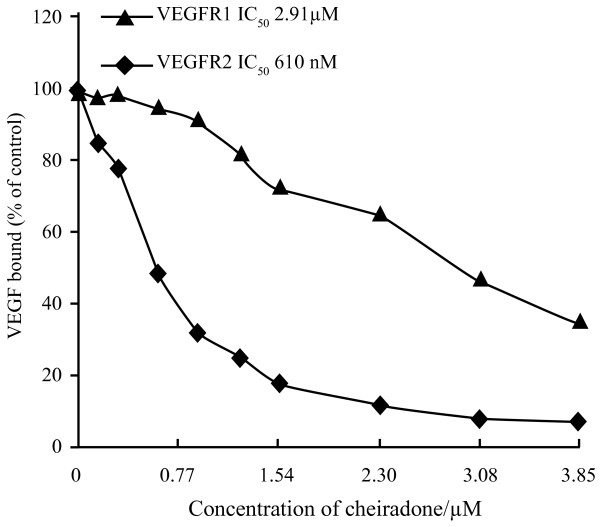
Cheiradone inhibits VEGF binding to VEGFR-1 and -2. Cheiradone was incubated with VEGFR-1 and VEGFR-2 in the presence of cheiradone (0–3.85 μM) and the binding of VEGF_165 _was measured as described above.

Cheiradone was found to specifically inhibit the binding of VEGF_165 _to VEGFR-1 and VEGFR-2 in a dose dependent manner with IC_50 _values of 2.9 ± 0.31 μM and 0.61 ± 0.14 μM respectively (Figure 1; Table 1). No significant inhibition of FGFR-1 and -2 was observed even at the highest concentration (328.20 μM) tested (data not included).

**Table 1:** A summary of the anti-angiogenic properties of cheiradone. IC_50 _values are The mean of three determinations.

**Experiment**	**IC_50 _value ± SEM (μM)**	
	**BAEC**	**HDMEC**

VEGF-R1 binding	2.9 ± 0.031	
VEGF-R2 binding	0.61 ± 0.14	
VEGF induced Proliferation	7.8 ± 1.2	7.4 ± 0.74
Effect on VEGF-induced wound healing	7.1 ± 0.77	6.50 ± .0.97
Effect on VEGF-induced migration	7.5 ± 0.92	5.20 ± 0.38
Effect on VEGF-induced tube formation	7.7 ± 1.5	6.0 ± 0.38
Effect on VEGF-induced invasion	6.3 ± 0.31	8.3 ± 1.0
Toxicity MTT	NS	NS
Toxicity Caspase-3	NS	NS

• NS = Overall inhibition is less than 50% and therefore IC_50 _values cannot be calculated.

### Cheiradone inhibited VEGF-induced EC proliferation

**Figure 2:**
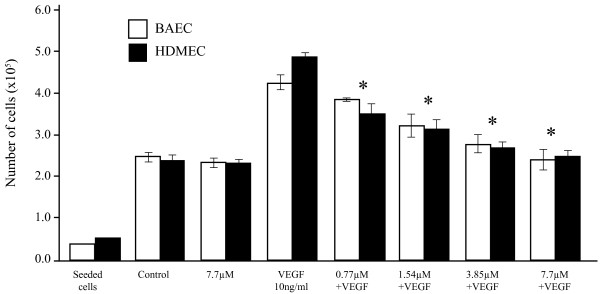
Cheiradone inhibited cell proliferation. Cells were added to a 6-well plate (seeded cells; BAECs 2 × 10^4 ^and HDMECs 3 × 10^4^) and the effect of cheiradone (0–7.7 μM) on VEGF_165 _(10 ng/ml in each case) induced proliferation was determined as described above. DMSO alone was added to the control and columns 3 and 4 represent cheiradone (7.7 μM) or VEGF alone. Values which differ significantly (p < 0.05) from VEGF alone are indicated by *.

Cheiradone was tested to evaluate its effect on cell proliferation in the presence of VEGF, EGF and FGF-2. A concentration-dependent inhibition of VEGF-stimulated BAEC and HDMEC proliferation with IC_50 _values of 7.4 ± 0.74 and 7.8 ± 1.2 μM respectively (p < 0.005) (Figure 2; Table 1) was observed. However, no significant inhibition of FGF-2- and EGF-triggered cell proliferation was observed (data not included).

### Cheiradone inhibited VEGF-induced EC Migration

**Figure 3:**
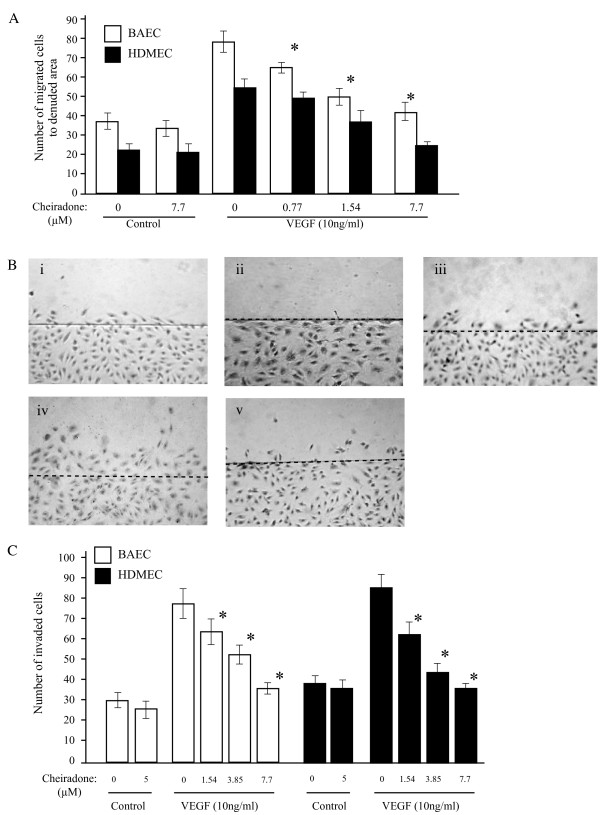
**C**heiradone inhibited cell migration. **(A) **A cell monolayer was wounded as described and recovery was assessed in the presence of increasing concentration of cheiradone (0–7.7 μM) and VEGF_165 _(10 ng/ml in all cases). Controls without VEGF contained either DMSO or cheiradone (7.7 μM). Values significantly different from VEGF alone (p < 0.05) are shown by *. (**B**) Representative photomicrographs show the results for HDMEC: **i **control, DMSO alone; **ii **time zero control; **iii **cheiradone 7.7 μM; **iv **treated with VEGF_165_; 10 ng/ml, **v **cheiradone + VEGF. (**C**) The effect cheiradone on VEGF-induced cell migration in a three dimensional Boyden chamber assay. Cells were added to the upper chamber and the cheiradone (0–7.7 μM) with and without VEGF (10 ng/ml) were added to the lower part. The total number of migrated cells to the lower chamber was counted as described above. Controls without VEGF contained either DMSO or cheiradone (5 μM). Values significantly different from VEGF alone (p < 0.05) are shown by *.

The effect of cheiradone on the migration of ECs was analysed using both two- and three-dimensional cell migration assays. In the two-dimensional assay, a wound-healing model was used to assess the migratory behaviour of BAECs and HDMECs (Figure 3). In the VEGF treated control group, significant wound healing was found 24 h after the cell monolayer was wounded with a sterile razor blade (Figure 3Biv; results shown for HDMEC). No significant inhibition was observed on non-stimulated wound recovery (Figure 3Biii) However, VEGF- stimulated HDMEC and BAEC migration was inhibited after 24 h in a dose dependent manner by cheiradone with IC_50 _values of 6.5 ± 0.97 and 7.1 ± 0.77 μM respectively (p < 0.05; Figure 3A and 3Bv). In a three-dimensional cell migration assay, BAECs and HDMECs treated with VEGF showed 2.8 and 2.5 fold increase in migration to the lower chamber compared to non-treated cells respectively (Figure 3C). Cheiradone was found to significantly inhibit (p = 0.001 in each case) VEGF-induced BAEC and HDMEC migration with IC_50 _values of 7.5 ± 0.92 and 5.2 ± 0.38 μM respectively.

Cheiradone had no effect on FGF-2 or EGF stimulated migration in the concentration range used (results not shown).

### Cheiradone inhibited VEGF-induced EC tube formation

**Figure 4:**
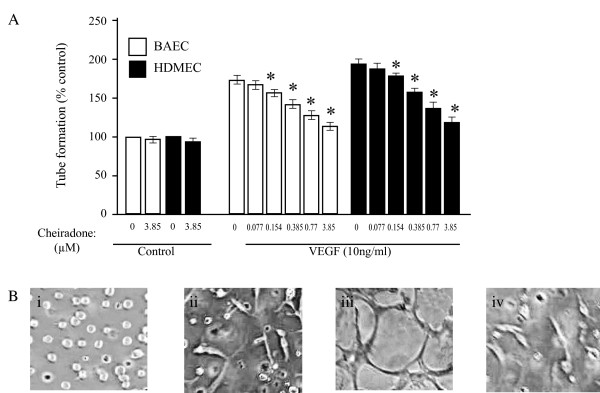
(**A**) Cheiradone inhibits EC differentiation into capillary like structures on Matrigel. Cells were treated with either cheiradone (control, 0–3.85 μM) or cheiradone (0–3.85 μM) with VEGF (10 ng/ml). Values significantly different from VEGF alone (p < 0.05) are shown by *. (**B**) Representative photomicrographs show; **i **HDMEC, **ii **HDMEC treated with cheiradone; 7.7 μM, **iii **HDMEC treated with VEGF 10 ng/ml, **iv **HDMEC treated with VEGF and cheiradone, 7.7 μM. Experiments were performed and the number of closed tubes was determined as described above. Results are the mean of three experiments. Incomplete tube formation was noted in the presence of cheiradone (**ii**) but extensive tube formation with VEGF (**iii**). In the presence of cheiradone the effect of VEGF was abolished (**iv**).

To examine the role of cheiradone on EC differentiation into vascular structures *in vitro*, tube formation of BAECs and HDMCs on Matrigel was assessed. When these cells were stimulated with VEGF, elongated tube-like structures were formed and the process was inhibited in a dose dependent manner by cheiradone (Figure 4A). Cheiradone reduced the width and length of VEGF-induced HDMEC and BAEC tubes with IC_50 _values of 6.0 ± 0.38 and 7.7 ± 1.5 μM respectively (p < 0.005; Fig 4Bi–iv, results are shown for HDMEC). No significant effect of cheiradone was seen on non-stimulated tube formation (Figure 4Bii).

### Cheiradone inhibited cell invasion

**Figure 5:**
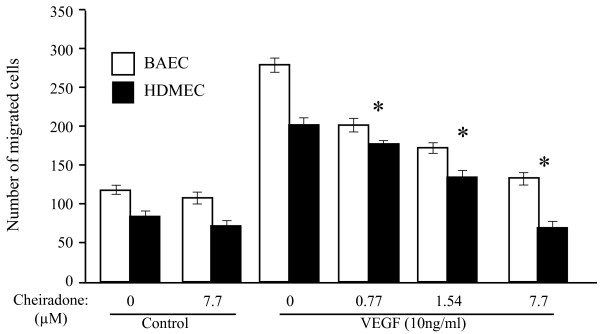
Cheiradone inhibits cell invasion. The effect of cheiradone on BAEC, and HDMEC cell invasion was studied using the chemoinvasion assay. Cells (1.7 × 10^4^) were added to the Matrigel coated upper Boyden chamber and cheiradone (0–7.7 μM) (control) or cheiradone with VEGF (10 ng/ml) was added to the lower chamber. Cell invasion was measured after 24 h and assessed as described above. Values significantly different (p < 0.05) from VEGF alone are shown by *.

The effect of cheiradone on cell invasion was analysed using a Transwell Boyden chamber system coated with reconstituted growth factor-reduced Matrigel. BAECs or HDMECs were allowed to invade the lower chamber in the presence and absence of VEGF and cheiradone. A statistically significant increased in cell invasion was observed in VEGF treated HDMECs (2.2 fold, p = 0.002) and BAECs (3.3 fold, p = 0.001) (Figure 5). Cheiradone showed dose-dependent inhibition of VEGF stimulated cell invasion of HDMEC and BAEC with IC_50 _values of 8.3 ± 1.0 and 6.3 ± 0.31 μM respectively (p < 0.05).

### Cheiradone inhibited angiogenesis in the CAM assay

**Figure 6:**
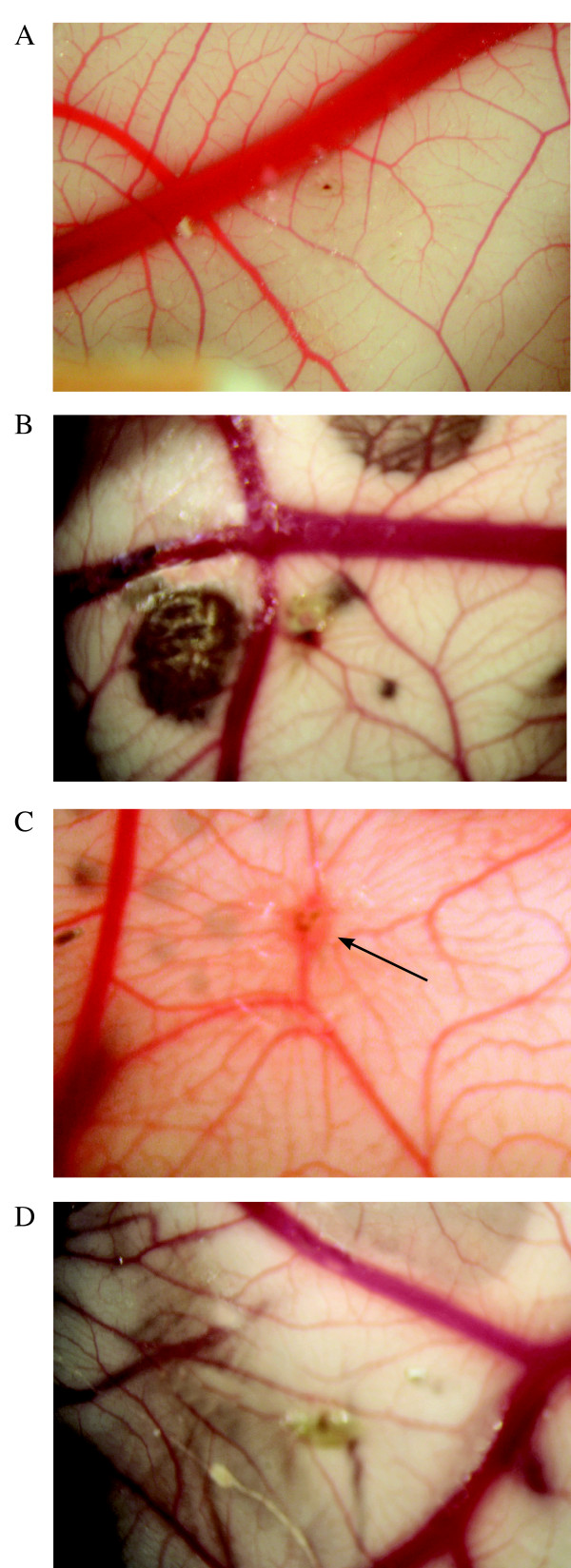
Cheiradone inhibits *in vivo *angiogenesis in the CAM assay. (**A**) control, methylcellulose alone showed no evidence of angiogenesis or inflammation (**B**) cheiradone (10 μg) failed to induce blood vessel formation (**C**) VEGF, a network of new blood vessels was established (see arrow) at the point of application. (**D**) cheiradone (10 μg) + VEGF inhibited vessel formation. There was no evidence of an inflammatory response in any preparation. Representative photomicrographs are shown. Magnification ×50.

The above *in vitro *data suggests that cheiradone inhibits several steps of angiogenesis *in vitro*. Therefore, we analyzed its effect on *in vivo *angiogenesis using the CAM assay. After exogenous stimulation of angiogenesis with VEGF_165_, significant new blood vessel growth was observed towards the stimulus after 6 days (Figure 6C, m = 3, n = 5). There was no significant angiogenic response to cheiradone alone (Fig 6B, m = 0.5, p = 0.0875, n = 5). VEGF_165_-induced blood vessels formation was completely abolished in the presence of cheiradone (m = 0, n = 5, p = 0.0001) (Figure 6D). There was no evidence of an inflammatory response with cheiradone alone (Fig 6) or in the control (Figure 6A).

### Cytotoxicity study

**Figure 7:**
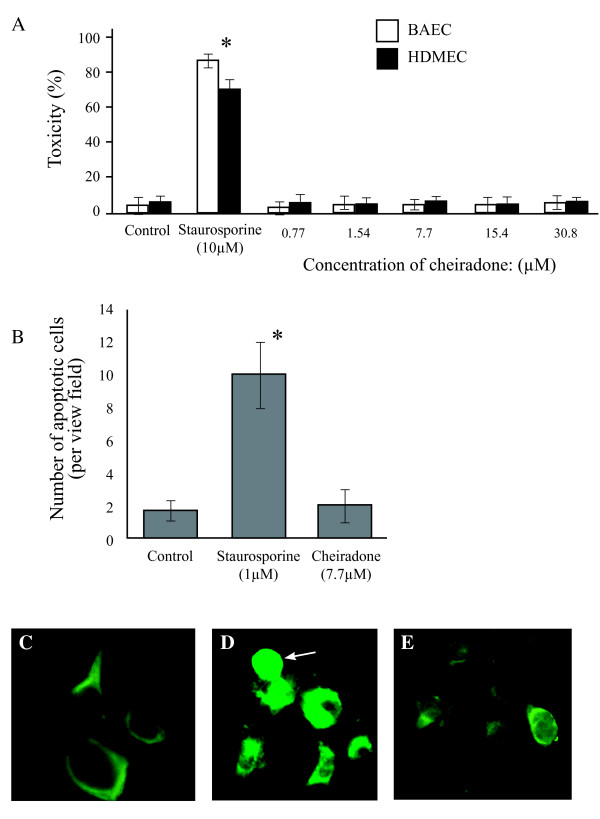
Investigation of the cytotoxic effect of cheiradone on BAEC and HDMEC viability. The cytotoxic effect of cheiradone was determined using (**A) **MTT assay and (**B**) Active-caspase-3 apoptosis assay. Cells were incubated with cheiradone and staurosporine (1.4 μM) for 24 h. Values significantly different from the control (DMSO) are shown by *. Immunofluorescence photomicrographs for HMDEC were taken as described above and show. (**C**) control, DMSO (**D s**taurosporine, the arrow indicates a positively stained apoptotic cell and (**E**) cheiradone.

No significant cytotoxicity was found at the tested concentrations of cheiradone (Figure 7A), whereas staurosporine induced a noticeable cytotoxic effect in the MTT (Figure 7B) and immunofluorescence (Figure 7D) assays.

## Discussion

Sesterterpenes are naturally occurring polyisoprene compounds widely distributed in plants and animals. There is growing interest in these molecules as potential disrupters of protein-protein interactions [25] since many protein interfaces are characterised by extended, flat surfaces and a number of small molecules which interfere with protein-protein binding have been identified [26]. In addition, members of the sesterterpene family have moderate antibacterial activity against *Mycobacterium tuberculosis *strain H(37)Rv, inhibit DNA replication [27], are cytotoxic against tumour cell lines [28] and have potent antiplasmodial properties [29].

During angiogenesis, nascent blood vessels grow by sprouting from the existing vasculature by a cascade of events including degradation of the basement membrane, EC migration, proliferation and tube formation [30]. VEGF exerts its angiogenic effect by binding to high affinity receptors on EC. In addition other growth factors, FGF-2 and EGF and their corresponding receptors are associated with angiogenesis [31].

In this study, we demonstrated that cheiradone inhibited multiple steps of VEGF-induced angiogenesis *in vitro *and *in vivo*. VEGF is the main regulator of angiogenesis and elevated levels have been reported in pathological conditions. The binding of VEGF to high affinity tyrosine kinase receptors such as VEGFR-1 & 2 activates VEGF-dependent signalling cascades which initiate the early events of angiogenesis (cell proliferation and migration from the lumen of existing vessels). Our *in vitro *inhibition data showed that cheiradone appeared to inhibit EC proliferation and migration with IC_50 _values in the range 5.2–7.8 μM. In the later stages of angiogenesis, ECs differentiate into tubular like structures that eventually form the lumen of the new vessel. The *in vitro *Matrigel tube formation assay showed that cheiradone inhibited VEGF-induced tube-like structures at low concentrations. We also demonstrated that cheiradone completely inhibited angiogenesis *in vivo *using the CAM assay. Therefore, cheiradone appears an effective antagonist of angiogenesis. Cheiradone was equally effective at inhibiting angiogenesis in both large vessel-derived (BAEC) and small vessel-derived cells (HDMEC; the *in vivo *target of angiogenic modulators).

Binding studies with VEGFR-1 and -2 showed significant inhibition of VEGF binding in the presence of cheiradone, with stronger inhibition of VEGFR-2. When cells were pre-incubated with cheiradone, and the cheiradone removed prior to addition of VEGF a significant inhibition of cell proliferation was still observed, indicating that cheiradone was not interacting directly with VEGF. Instead cheiradone competed with VEGF for binding to a VEGF receptor. Both VEGFR-1 and -2 contain extracellular, juxtamembrane and tyrosine kinase domains. We have no evidence which domain cheiradone binds to and are currently investigating the tyrosine kinase activity of the VEGF receptors in the presence of the inhibitor. An additional mechanism by which cheiradone can affect VEGF-induced angiogenesis is by regulating the expression levels of VEGFR-1 and/or VEGFR-2. We are currently investigating the interaction of cheiradone with the receptors on endothelial cells.

Semino *et al*., [32] have developed an *in vivo *model of angiogenesis in the presence of interstitial flow. They propose a two step model of angiogenesis requiring initial activation by VEGF and subsequent maturation of the new blood vessel on exposure to EGF. Cheiradone would be an ideal molecule to test this model since it has no activity against EGF. *In vivo*, VEGFR-1 is constitutively expressed in the blood vascular system while VEGFR-2 is down regulated but is over expressed in angiogeneic endothelial cells and after hypoxia [33]. We have shown that cheiradone is more active against VEGFR-2 and may therefore be a more specific molecule for targeting angiogenic blood vessels in diseases such as cancer.

In addition, cytotoxicity studies showed that cheiradone had no adverse effects at concentrations greater than those used in the present study.

The advantage of cheiradone over existing VEGF inhibitors is that it does not remove VEGF from the system and VEGF activity may be modulated by varying the concentration of cheiradone.

## Conclusion

We have demonstrated that cheiradone, a naturally occurring sesterterpene inhibits VEGF-induced angiogenesis by competing with VEGF for VEGFR-1 and -2. There was no activity against FGF-2 or EGF. Further study of the structural relationships of cheiradone and its activity may provide a basis for designing VEGF receptor antagonist with enhanced inhibitory potential and improved specificity.

## Methods

### Test compound

**Figure 8:**
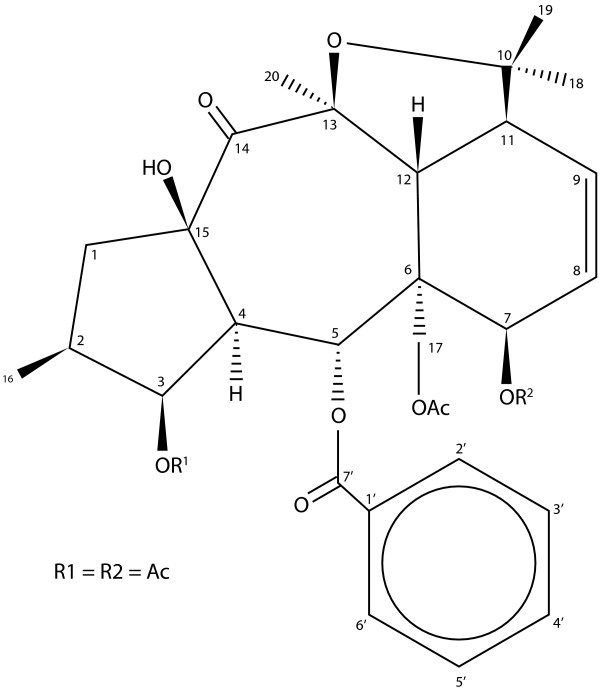
The structure of cheiradone.

Cheiradone (Fig 8; RMM 613) was extracted and purified from *Euphorbia cheiradenia *Boiss. Full chemical characterisation and purification is detailed elsewhere [24].

### Materials

Matrigel (Becton Dickinson, UK), FGF-2, goat anti-active caspase-3 antibody (R&D System), VEGF_165 _(Apollo Cytokine Research, Cambridge, UK), EGF, VEGFR-1 and -2 and FGFR-1 and -2, anti-FGF-2 and anti-VEGF antibodies (Santa Cruz Biotechnology, Heidelberg, GDR), ABTS peroxidase substrate kit (Vector, UK), Transwell chamber system, culture plates and flasks (SLS, UK), anti-goat Alexa flour 488 conjugated green fluorescence dye and other chemical of commercial grade were purchased from Sigma (Poole, UK).

### Cell Culture

Human dermal microvascular endothelial cells (HDMECs) and the appropriate medium were purchased from TCS Cellworks (Buckingham, UK) and were cultured and maintained according to the supplier's instructions. Bovine aortic endothelial cells (BAECs) were from an established large vessel primary cell culture obtained and characterised as described previously [34]. They were routinely cultured in Dulbecco's modified Eagles medium (DMEM) containing varying concentrations of foetal calf serum (FCS). All cells were used between passages six to nine.

### Cell proliferation assay

Cells were seeded in triplicate at a concentration of 2.5 × 10^4^/ml, in 2 ml of complete medium in 6-well plates. After attachment (24 h), medium was replaced with serum poor medium (SPM), containing 2.5% foetal calf serum in which the cells grew at a significantly reduced rate. Growth factors FGF-2 and EGF (25 ng/ml), and VEGF_165 _(10 ng/ml) with and without the test compound at different concentrations was added and cells incubated for a further 72 h. Control wells were treated with 5 μl DMSO. Concentration ranges of test compounds and pre-incubation times were based on pilot studies. MTT and immunofluorescence studies using active caspase-3 as a measure of apoptosis confirmed that test compounds were not cytotoxic at the concentrations used (see below). After 72 h, cells were washed in PBS, detached in 0.05%w/v trypsin in PBS, and counted on a Coulter counter (Coulter Electronics, Hialeah, FL) set to a threshold of 30 μm. Experiments were performed at least twice in triplicate wells and significance was determined by the Student *t *test. A representative example is shown.

### Cell migration assay

Cell migration was examined *in vitro *using a Transwell chamber system with 8.0 μm pore polycarbonate filter inserts (TSL, UK). The Transwell insert was coated overnight with 0.1%w/v gelatine, and air-dried. Cells (1 × 10^5^) were placed in the upper part of the filter and test compounds at different concentrations with and without growth factors were added to the lower part in SPM. Cells were incubated at 37°C for 16 h. After fixation (4% paraformaldehyde), and staining (Geimsa), cell migration in duplicate wells was determined by counting cell numbers on the lower surface. Experiments were performed at least twice and a representative example is shown. Significance was determined by the Student *t *test.

### Wound healing assay

Cells (6 × 10^4^/ml) were added to a Thrermanox cover slip in a 24-well plate in complete medium and incubated for 24–48 h. When confluent, the medium was replaced with DMEM containing 0.1% FCS and incubated for a further 48 h. Cover slips were washed (PBS, ×3), wounded with a sterile razor to produce a straight edged cut and washed in PBS to remove dislodged cells. Cover slips were added to a fresh 24-well plate in 0.1% FCS and incubated with VEGF_165 _or the other growth factors and a range of concentrations of test compound for 24 h. Under these conditions there was negligible proliferation but measurable migration. Slides were fixed in ethanol (100%), stained with methylene blue and photographed. For each slide, 10 fields of view (2 mm × 1.45 mm) were counted at random. Each experiment was performed at least twice and significance was determined by the Student *t *test.

### Cell differentiation assays in Matrigel

Cells (1.0 × 10^6^/ml) were mixed with an equal volume of Matrigel at 4°C. Aliquots (45 μl) were added to the wells of a 48-well plate and allowed to polymerise (1 h) when 500 μl of microvascular endothelial cell medium containing VEGF_165 _or the other growth factors, with or without the test compound was added. The cells were incubated for 24 h at 37°C then fixed in 4% paraformaldehyde (5 min), washed in cold ethanol and air dried. Cells were stained with Geimsa (30 sec), air dried and photographed. Ten random fields were selected and the number of closed tubes counted.

All experiments were performed in triplicate and repeated at least twice and significance was determined by the Student *t *test.

### Chemoinvasion assay

A Transwell cell culture chamber with 6.5-mm-diameter polycarbonate filters (8-μm pore size) were coated with 30 μg/ml Matrigel. Cells (1 × 10^5^) were added to the upper chamber suspended in an appropriate medium. The medium containing a range of concentrations of test compound was added to the lower chamber in the presence and absence of VEGF_165 _or the other growth factors. After 24 h incubation at 37°C, the medium from the lower chamber was removed, cell fixed (4% paraformaldehyde) and stained. The number of cells invaded to the lower chamber through the Matrigel was counted under phase-contrast microscopy. Each invasion experiment was performed in triplicate and repeated at least twice. Statistical significance was determined using the Student *t *test.

### Binding assay

Competition between growth factors, their cell surface receptors and test compounds was assessed as described previously [35]. A 96-well plate was coated overnight with growth factors (2 μg/ml of VEGF_165 _or FGF-2) and blocked with 1% BSA in PBS containing 0.05% Tween-20. The test compound was separately pre-mixed with soluble receptors VEGFR-1, VEGFR-2, FGFR-1 or FGFR-2 (2 μg/ml in each case) for 2 h and added to the plate and incubated for a further 2 h. The plate was washed (×3 with PBS-Tween-20) and incubated with anti-VEGF or anti-FGF-2 antibody (1:500 in PBS-Tween-20) for 45 min, washed and incubated with horseradish peroxidase conjugated goat anti-IgG (Santa Cruz, 1:1000) for a further 45 min. After washing (×3), ABTS peroxidase substrate (Vector, UK) was added and the absorbance read at 405 nm. IC_50 _values were calculated from the data using the EZ-Fit enzyme kinetic software (Perella Scientific Inc., Amherst, USA).

### Chick chorioallantoic assay

The angiogenic activity of cheiradone was determined using the semi-quantitative chick chorioallantoic assay (CAM) as described previously [36]. To expose the CAM a window was created in the shells of 4 day-old chicken eggs. On day 8, a 2 mm^3 ^methycellulose pellet (5 μl of 1% sterile methylcellulose; 400 centipoise, Sigma UK) containing no additions (control), the test compound (10 μg) with and without VEGF (100 ng) were applied to the membrane. The resultant angiogenesis scored on day 14 as 0- negative; 0.5- change in vessel architecture; 1- partial spokewheel (1/3 of circumference exhibits directional angiogenesis); 2- spokewheel; 3 or greater-strong and fully spokewheel. This approach enabled calculation of an accumulated response in each group. To photograph the membrane, 2 cm^3 ^of a 50% emulsion of aqueous paraffin oil containing 2% Tween-80 was injected at the site of application and photographed using a Leitz dissecting microscope. Each experiment was performed five times and statistical significance was determined by the Mann-Whitney U test and the data is expressed as a median value (m).

### Toxicity Assays

Cheiradone toxicity was determined using MTT and active caspase-3 assays. BAECs or HDMECs (7.5 × 10^3^) were seeded in a 96 well plate and incubated for 4 h to allow cell adhesion. Cheiradone or staurosporine, an inducer of active caspase-3 and therefore, of apoptosis (1 μM) was added to the wells. Control cells were treated with PBS and the plate was incubated at room temperature for 72 hours. MTT reagent (10 μl) was added followed by incubation at room temperature for 2–4 h. When a purple precipitate was visible, detergent reagent (100 μl) was added to the plates and incubated at room temperature for 2 h in the dark. Absorbance was measured at 570 nm using a microplate reader.

In the apoptosis assay, HDMECs or BAEC (4 × 10^4^/ml) in complete medium were added to the chambers slide and allowed to adhere for 24 h. Cheiradone (8.2 μM) or staurosporine (1 μM) were added to all wells except control (PBS) and incubated for 24 hours. Wells treated with staurosporine were immediately washed (PBS) and fixed (4% paraformaldehyde) when cell morphology became round (2–4 h). After washing and fixing, cells were permeablized (0.1% Triton X-100; 10 min), washed (×5 ~ 5 min each), air dried and blocked with 1% BSA in 1:50 TBS Tween for 1 h at room temperature. Cells were incubated with goat anti-active caspase-3 (R&D system, UK; 1% BSA in TBS Tween) for 1 h. The plates were incubated with anti-goat Alexa-Flour 488 conjugated green fluorescent dye for 1.5 h at room temperature. Ten random homogeneous fields were viewed, and photographed.

## Authors' contributions

SH carried out the angiogenic assays.

JG, NA and MS participated in the design of the study and preparation of the manuscript. MM, MC and AE isolated and characterised the cheiradone.

SM carried out the experiments on tube formation DW performed the CAM assay. All authors read and approved the manuscript.
